# An Inter-comparison of Three Heat Wave Types in China during 1961–2010: Observed Basic Features and Linear Trends

**DOI:** 10.1038/srep45619

**Published:** 2017-03-31

**Authors:** Yang Chen, Yi Li

**Affiliations:** 1 Chinese Academy of Meteorological Sciences, State Key Laboratory of Severe Weather, Beijing, 100081, China; 2 Public Meteorological Service Center, China Meteorological Administration, Beijing, 100081, China

## Abstract

Using observed daily temperatures in China, three independent types of heat waves (HWs), including daytime HWs, nighttime HWs, and compound HWs (with both extreme daily maxima and minima), were defined. Different types of HWs showed distinctive preferences in occurrence locations and timing. However, spatial patterns of accompanying relative humidity were generally independent of categorization, except for closer association of nighttime events with high humidity level. Compound HWs and nighttime HWs experienced significant increases in frequency, participating days, mean duration, intensity and areal extent. Conversely, significant decreasing trends of above indicators prevailed in daytime HWs, especially in central-eastern China. Tendency of relative humidity changes didn’t vary with HW types. Instead it caused an interesting phenomenon that dry HWs in the west became more humid and humid events in the east got dryer, as manifested most obviously in compound type. Thorough comparisons highlight the evolutionary dominance of HW types. Specifically, previously-dominating independent daytime HWs have been increasingly replaced by independent nighttime events in central-eastern China, and by compound HWs in southern China. That’s the very reason for negative trends of independent daytime HWs in eastern China, even in a warming climate.

During the last decade, severe heat waves (HWs) have been frequently registered in many parts of the world. These high-profile HWs exerted adverse effects on normal functioning of society, human health, agriculture, and ecosystem. For instance, in 2003, the European continent experienced an unprecedented HW characterized by extraordinary long duration, excessive high temperature and vast spatial scale^1^. This devastating heat wave took a heavy toll on human lives (at least 50,000 deaths)^2^. Exact identification of HWs and precise comprehension of their changes are prerequisites for developing mitigation and adaptation strategies against heat-related consequences.

A HW is generally regarded as an event that exceeds prescribed temperature thresholds over a few days. Concrete definitions, however, vary greatly among literatures that focused on different aspects of HWs^3^. In the climate science community, a HW usually refers to a period of at least 3 days with extreme maximum temperature (Tmax)^4^. Epidemiologists attach greater importance to daily minimum temperature (Tmin) when investigating heat wave-related mortality^5^,^6^. Relative humidity is another variable often involved in HW definition, because high humidity may aggravate impacts of HWs on human thermoregualtion^7^,^8^,^9^.

For temperature extremes in China, numerous studies focused on individual warm days or nights, with their duration less emphasized^10^,^11^. Thus, compared to evaluations of HWs in the US, Europe and Australia, studies assessing HWs across China are much fewer, the majority of which were primarily concerned about occurrence frequency^12^,^13^,^14^,^15^. However, basic features and changes of other equally-important aspects, such as duration, areal extent and intensity, remain substantially under-assessed. So a synthesized matrix containing multiple indicators is desirable to systematically quantify HWs in China. Moreover, for both scientific literatures and operational practices, previous definition of heat waves in China only considered extreme Tmax, so identified events were more indicative of scorching conditions during daytime^13^. Yet, a more precise and practical evaluation should distinguish different types of HWs, due to their differing impacts^16^. For example, a “daytime heat wave” (only extreme Tmax) usually induces heat strokes, blight of crops, rising risks of wildfire and great burdens on water-electricity supply^17^,^18^. Fortunately, accompanying cool nights provide adequate time for human and ecosystem to recover^6^,^16^; while, regardless of daytime situation, catastrophic failures in human thermoregulation mainly occur during “nighttime heat waves”^5^,^19^. “Compound HWs” with simultaneously elevated Tmax and Tmin are undoubtedly most devastating. Some notorious HWs, which claimed thousands of lives and billions of economic losses, were typical of compound HWs^20^,^21^,^22^. In addition to different impacts, responsible mechanisms may also vary significantly among different HW types^16^,^23^,^24^.

Some recent studies in the Europe and the US began to realize the necessity of separately assessing diverse HW types^3^,^25^,^26^. However, the categorization in these studies was performed by delimiting single temperature variable (Tmax or Tmin). Such single-variable based definition possibly fails to group HWs properly, because identified HWs may be interchangeable among diverse types. For example, a daytime event is usually accepted when Tmax is above 90^th^ percentile for several days^26^,^27^. Whereas if Tmin also exceeds its 90^th^ percentile during some or all these days, this event may actually be (at least partly) a compound HW, rather than a well-defined independent daytime event as expected. Thus, derived climatological characteristics and estimated trends for independent daytime events actually contain mixed signals of compound events and daytime events. Such smeared estimations would easily distract policy-makers and general public from efficient precautions. It is therefore suggested to delimit both Tmax and Tmin to achieve precise catergorization^23^.

Although studies on definitions of multiple HW types have begun to emerge recently, very few of them have conducted inter-comparisons among diverse types. This is especially true for heat wave studies in China. This study therefore aims to narrow the aforementioned gaps. Specifically, focusing on mainland China, this study will reveal and compare observed basic features of daytime HWs, nighttime HWs and compound HWs during 1961–2010. Then, linear trends of multiple aspects for different HW types would be estimated and compared. This observational analysis serves as a basic yet imperative step towards further physical interpretation, attribution and projections of heat waves.

The observational data, methods, definitions and indices would be introduced in “Data and Method” Section. Main analyses and comparisons would be presented in “Results” Section, followed by a brief summary and some discussions in “Conclusions and Discussions” Section.

## Results

### Basic features

**Figure 1:**
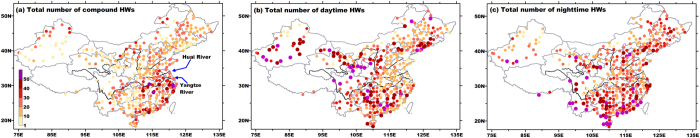
Accumulated frequency of different heat waves (HWs) in China during 1961–2010. (**a**) for compound HWs; (**b**) for daytime HWs; (**c**) for nighttime HWs. The frequency values above 40 are highlighted by larger dots. Three black curves in the basemap from north to south represent the Yellow River, the Huai-River and the Yangtze River respectively, as labeled in (**a**). This figure was created by Surfer 7.0 (http://www.goldensoftware.com/products/surfer).

With considerable emphasis placed on linear trends, whether and how climatological features vary with HW types have not yet been revealed in existing studies. Figure 1 shows accumulated occurrences during 1961–2010. In general, compared to independent daytime or nighttime events, compound events were less frequently observed. Interestingly, the occurrence of different HWs exhibited obvious location preferences. Most compound HWs appeared in eastern China (east of 115°E), especially across mid-lower reaches of the Yangtze River. Coastal areas in South China also suffered from frequent compound HWs during the past few decades. Both of these two regions are economically developed and densely populated, so recurrent compound HWs could result in great morbidity/mortality and economic losses, owing to high exposure to extremes there. In contrast, the majority of daytime HWs concentrated in the western part (west of 115°E), particularly in Northwest China. The nighttime HWs occurred preferably in southern parts of China.

**Figure 2:**
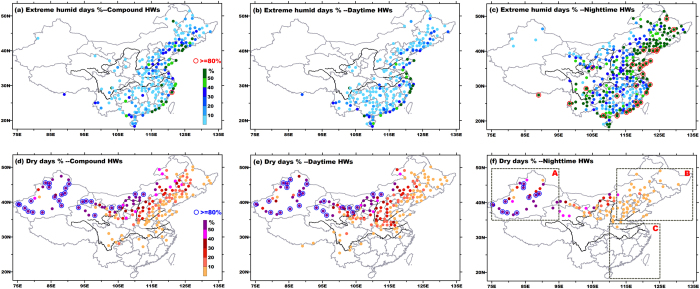
Percentages of humid and dry days during heat waves of each type. Percentages of extreme humid days (daily mean relative humidity ≥80%) in total participating days are displayed in (**a**–**c**), in which stations with percentages above 80% are highlighted by red circles. Percentages of dry days (daily mean relative humidity <40%) in total participating days are displayed in (**d**–**f**), in which stations with percentages above 80% are highlighted by blue circles. The grey rectangles in Fig. 2e labels typical regions shared by all the three types, which respectively record frequent dry days (A), frequent humid days (C), and both dry and wet days (B). This figure was created by Surfer 7.0 (http://www.goldensoftware.com/products/surfer).

Accompanying relative humidity anomaly can exacerbate impacts of HWs. On one hand, during extreme hot periods, the mortality increases exponentially with relative humidity once exceeding 60%^28^; on the other hand, during severe droughts, HWs with low relative humidity (lower than 40%) may aggravate damages to agriculture^29^. In order to measure humidity levels during HWs, percentages of humid and dry days in total participating days were calculated. Figure 2 presents a generally consistent spatial pattern among diverse types, with dry events mainly observed in Northwest China and humid events mainly detected in eastern and southern China. So in nature, the spatial dependence of dry and humid HWs seems independent of HW types. Instead, it is essentially determined by local prevailing climate conditions, i.e. arid climate in Northwest China and moist monsoonal climate in eastern China^30^. Particularly, Fig. 2c and f clearly reveal that nighttime HWs tend to be accompanied by sharply heightened humidity, and behave more typical of “humid heat wave”^16^. During night, abundant atmospheric moisture inhibits outgoing long wave radiation and emanates downward long wave radiation, facilitating the establishment of warm environment near the surface. Likewise, resultant widespread cloudiness and possible precipitation reduce the chance of extreme Tmax in the next day, so independent nighttime events form.

**Figure 3:**
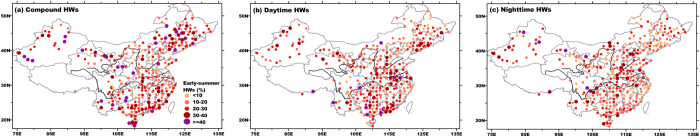
Percentages of early summer heat waves in each type. (**a**) for compound HWs; (**b**) for daytime HWs; (**c**) for nighttime HWs. The percentages above 30% are highlighted by larger dots. The early summer heat waves refer to the events with at least half participating days appearing before 20 June. This figure was created by Surfer 7.0 (http://www.goldensoftware.com/products/surfer).

The timing of HWs is also an important but little-reported attribute^8^. Most susceptible people died during early-summer HWs, because they have few opportunities to be fully prepared for sudden extreme hot conditions during early summer stage^31^. Though the vital influences of HW timing have been reinforced by epidemiologists, regions vulnerable to frequent early-summer HWs remain to be confirmed. As revealed in Fig. 3a, about 40–50% compound HWs in Northeast China were early-summer events. In Northwest China and Southwest China, early-summer compound HWs also accounted for reasonably large percentage. For daytime HWs, early-summer events were mainly clustered in the Yangtze-Huai River Valley and South China. In most parts of China, by contrast, nighttime HWs tended to occur much later (Fig. 3c).

### Linear trends estimation

**Figure 4:**
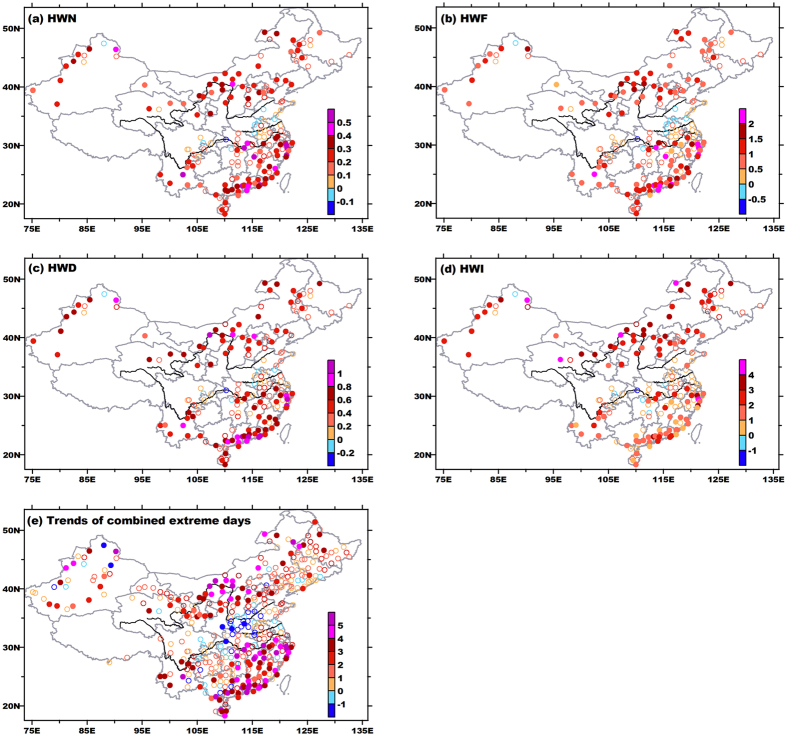
Linear trends of compound heat waves in (**a**) heat wave occurrences (HWN), times decade^−1^; (**b**) participating days (HWF), days decade^−1^; (**c**) mean duration (HWD), days decade^−1^; (**d**) heat wave intensity (HWI), °C decade^−1^; (**e**) percentage of combined extreme days (days with both extreme Tmax and Tmin), % decade^−1^. In (**a**–**d**), trends are shown at a station only when at least 20 years of events exist, and statistical significance is set at the 0.05 level. Significant and insignificant trends are denoted by shaded and blank dots, respectively. This figure was created by Surfer 7.0 (http://www.goldensoftware.com/products/surfer).

The occurrence frequency (HWN) of compound HWs increased in large parts of China, with the largest increases above 0.5 times decade^−1^ mostly observed in the Yangtze River Valley and South China (Fig. 4a). These two regions also experienced significant increases in participating days (HWF, Fig. 4b) and mean duration (HWD, Fig. 4c). Considering the mean duration being calculated as 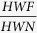, such lengthening trend suggests a larger magnitude in increases of HWF. The largest amplification of HWI (intensity, see Method Section) were found in North China, with quite a few stations registering trends above 3 °C decade^−1^, though these station experienced relatively smaller increases in duration (Fig. 4d). The HWI is designed to portray accumulated heat stresses during HWs, so it is a function of both duration and exceedance above the threshold. Thus, stronger amplification in intensity corresponding to smaller lengthening in duration may imply more drastically elevated Tmax and/or Tmin in North China.

**Figure 5:**
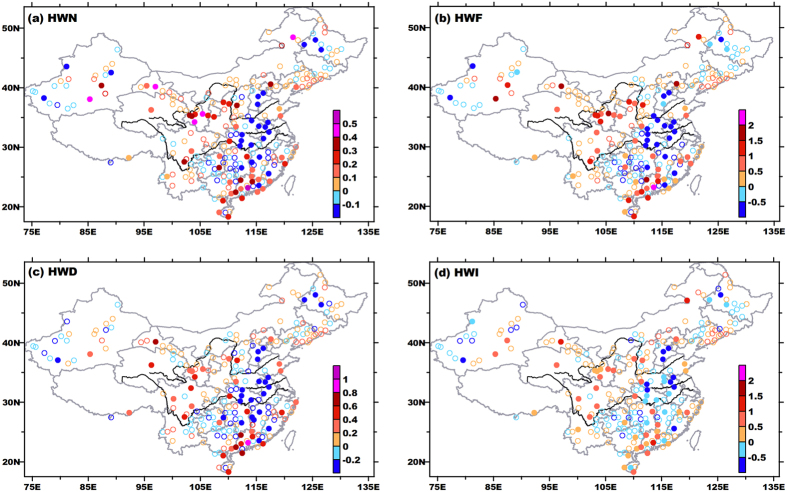
Same as inFig. 4, but for daytime heat waves. This figure was created by Surfer 7.0 (http://www.goldensoftware.com/products/surfer).

Changes of daytime HWs exhibited quite different spatial patterns (Fig. 5). Daytime HWs across central-eastern China became weaker, less frequent and shorter-lasting. Decreasing trends also appeared in parts of Northwest China, North China and Northeast China, where significant positive trends were detected in compound events. Other studies investigating Tmax-based HWs also reported decreases in HWN in the Huai-River Valley^13^,^32^, but the negative trends in their studies showed much smaller magnitude and less significance. This is because the trend in their studies actually involved mixed signals of significant/strong negative trends of daytime and insignificant/weak trends of compound HWs (Figs 4a and 5a). So in these traditional estimations, both magnitude and significance, or even signs of real trends for daytime events were contaminated and thus misleading. For daytime events, the number of stations with significant positive trends was much fewer than that in compound events, and these stations were distributed more scattering.

For each indicator of nighttime HWs, increasing trends constituted a nearly pan-China pattern. The stations with significant positive trends accounted for about 70% of total stations. The HWN, HWF, and HWD grew slightly faster in southern parts of China; while similar to the situation in compound events, nighttime HW intensity showed greater increasing rates in northern parts of China. Among these three HW types, the nighttime type is the only one that registered significantly increased trends in various aspects of HWs in the Huai River Valley.

**Figure 6:**
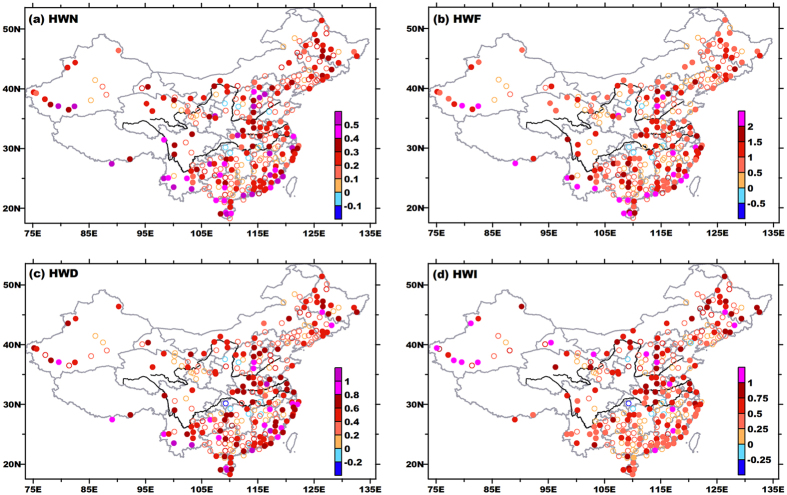
Same as inFig. 4, but for nighttime heat waves. This figure was created by Surfer 7.0 (http://www.goldensoftware.com/products/surfer).

The above comparisons among three types highlight that during the past few decades, HWs became more prevalent during nights (Figs 4 and 6), and daytime HWs were increasingly accompanied by nighttime expressions (Figs 4 and 5). The contrasting pattern between compound and daytime events indicates an enhanced coupling between extreme Tmax and Tmin in some regions. Such strengthened coupling can be evidenced by significantly increased proportion of combined extreme days (Tmax ≥ 90th percentile *and* Tmin ≥ 90th percentile) in total extreme days (Tmax ≥ 90th percentile *or* Tmin ≥ 90th percentile), as shown in Fig. 4e. Remarkably, in the Yangtze River Valley and northern parts of China, significantly negative trends of independent daytime events don’t really mean heat waves have less chance to appear in daytime. Instead, previous daytime events have increasingly evolved toward compound events, due to the above-mentioned enhanced coupling (Figs 4e and 5).

Variations of summertime mean temperatures (Tmean) may partly explain above changes of HWs. During 1961–2010, mean temperature during summer increased significantly across China, except for the Huai-River Valley (figure not shown, see Supplementary Fig. S1). All of Tmean, Tmax and Tmin showed much larger increases in northern parts of China, notably in Tmin. That validates the speculation about aforementioned disproportionate increases of intensity and duration in northern parts of China (Figs 4c,d and 6c,d). In spite of greater rises in magnitude in northern parts, larger increments of extreme days for both Tmax and Tmin mainly appeared in the lower reaches of the Yangtze River Valley and South China (Fig. S1d–f), accounting for larger trends for duration there. In entire mainland China, increase in hot nights far outdistanced (doubled or even tripled) the growth of hot days. Some of these incremental hot nights coupled with hot days to constitute or lengthen compound events, and others directly amalgamate to form more independent nighttime events. So both compound events and nighttime events showed significant increases in diverse aspects. Conversely, the Huai-River Valley recorded significant decreases of mean temperature, which mainly arose from significant cooling of Tmax in this region (figure omitted, see Supplementary Fig. S1b). Resulting significant reductions in hot days and weakened coupling between hot days/nights (Fig. 4e) are the direct reason for negative trends of independent daytime and compound events. Thus, the Huai-River Valley actually experienced fewer heat waves in the daytime, but saw more events during night (Fig. 6). So local people should place more emphasis on precautions against sultry weather during the nighttime, via both physiological and behavioral adjustments.

**Figure 7:**
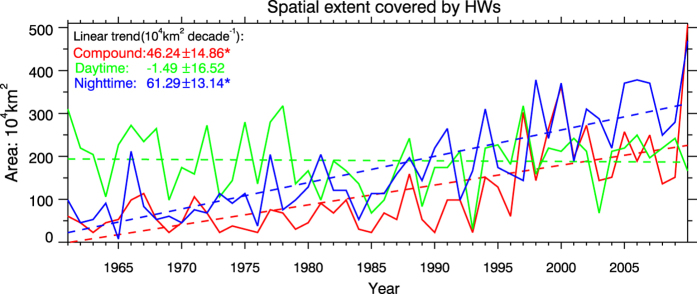
Temporal variations of spatial extent (10^4^ km^2^) covered by daytime heat waves (green solid line), nighttime heat waves (blue solid line), and compound heat waves (red solid line). Dashed lines of different colors represent linearly-fitted results with respective to each type. Estimated trends (10^4^ km^2^ decade^−1^) for each type are labeled in the upper-left corner, with *indicating their significance at the 0.05 level. The numbers behind “±” denote the 95% confidence intervals of estimated trends. This figure was created by IDL 7.1 (http://www.harrisgeospatial.com/ProductsandSolutions/GeospatialProducts/IDL.aspx).

Temporal variations of spatial extent also varied with HW types (Fig. 7). Generally, both compound and nighttime HWs affected broader areas, while the spatial coverage of daytime events decreased slightly. Before 1980, the daytime HW was the absolutely dominant type across China, validated by its spatial coverage even over total area of the other two types. Afterwards, the spatial extent of daytime events had shrunk gradually. Since 1961, the spatial coverage of nighttime HWs has showed a monotonically increasing tendency, with its expanding rate of 61.29 × 10^4^ km^2^ decade^−1^. For compound events, their spatial coverage grew very slowly (about 11.18 × 10^4^ km^2^ decade^−1^) before 1995; after 1995, however, the expansion dramatically accelerated with a linear trend of 56.99 × 10^4^ km^2^ decade^−1^. Thus, the average spatial extent of compound events during 1996–2010 reached 225 × 10^4^ km^2^, nearly accounting for one quarter of total area of mainland China. The broadening spatial scales of compound HWs and nighttime HWs accentuated an observed fact that these two types covered much vaster geographic areas where had never witnessed such kinds of events before. With respective to the period before 1980, the areal extent of compound HWs and nighttime HWs increased by 320% and 250% during the last decade. In particular, in 2010, the compound HWs swept more than half of China (about 480 × 10^4^ km^2^), so did nighttime HWs. The opposite variations in areal extent again highlight increasing transitions from daytime type toward nighttime-accentuated type.

**Figure 8:**
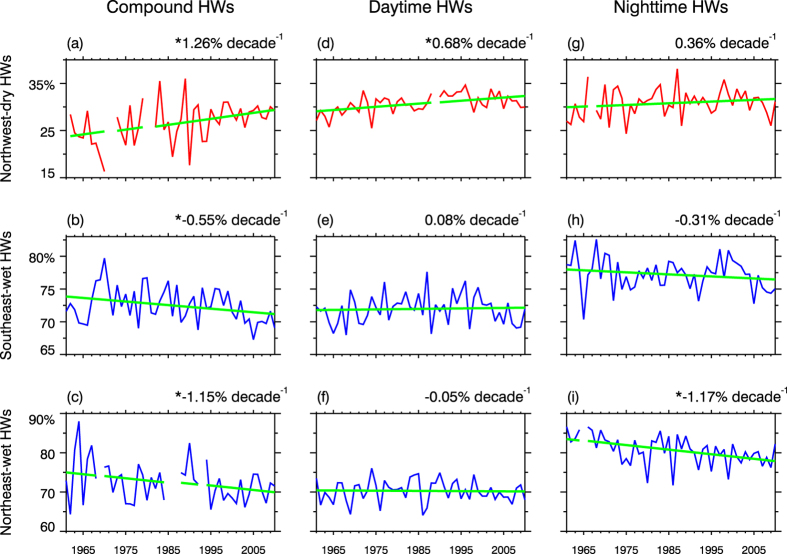
Temporal variations of mean relative humidity during heat waves of each type in diverse sub-regions. Each column corresponds to one heat wave type, and each row denotes one sub-region. The domain of each typical sub-region is labeled in Fig. 2e, with “Northwest” corresponding to region-A, “Southeast” corresponding to region-C, and “Northeast” corresponding to region-B. Dry (red) and wet (blue) HWs are judged by mean relative humidity during heat waves lower than 40% and higher than 60%, respectively. Estimated trends (% decade^−1^) are labeled in the upper-right corner, with *denoting their significance at the 0.05 level. Linearly-fitted results are shown in green lines. Break points in curves mean that no such kind of events was identified in corresponding years. This figure was created by IDL 7.1 (http://www.harrisgeospatial.com/ProductsandSolutions/GeospatialProducts/IDL.aspx).

The temporal variability of humidity during different HW types has been rarely reported in China. Criteria of mean relative humidity during HWs lower than 40% and higher than 60% were adopted to partition dry and wet HWs. Northwest China (region A in Fig. 2f) and Southeast China (region C in Fig. 2f) are typical dry-HW region and wet-HW region shared by all the three types. Northeast China (region B in Fig. 2f) seems a transitional region that recorded both dry and wet HWs. As indicated in Fig. 8, among diverse HW types, a coherent feature can be summarized as that dry HWs have been becoming wetter, and wet HWs have been getting drier. Obviously, such opposite variations between wet and dry events are not induced by type classification. Rather, above consistent variations among three types are embedded in the context of regional humidity changes that relative humidity increased in western parts and decreased in eastern parts (figure omitted, see Supplementary Fig. S2). Although the sign of trends for humidity were insensitive to HW types, the magnitude of relative humidity changes differed greatly among different types, punctuated by most significant and sharpest trends with compound HWs (Fig. 8a–c). Additionally, the opposite variations in humidity may enrich the diversity of HW impacts. For instance, dry HWs in northern parts of China used to impose great burdens on water-electricity supply and crop growth. With heightened humidity level, dry events would also be enabled to exacerbate their impacts on human health. Such enrichment of HW impacts superimposed by regional relative humidity changes would be manifested most obviously in compound events.

## Conclusions and Discussions

### Conclusions

In the context of global warming, there have been numerous studies investigating heat waves. Most of these studies focused on a single type of heat wave and estimated changes in occurrence frequency. Other important indicators, such as duration, intensity, areal extent, humidity and timing, were inadequately evaluated. Very few studies have separately assessed diverse types of heat waves and conducted systematic comparisons of their changes. These deficiencies are more prominent in studies on heat waves in China.

To fill above gaps, three types of heat waves (HWs), including daytime events (extreme Tmax only), nighttime events (extreme Tmin only) and compound events (extreme Tmax and Tmin), were defined and measured by diverse indicators. During 1961–2010, basic features and linear trends in multiple aspects of HWs varied among different HW types. The compound HWs were frequently observed in the Yangtze River Valley. About half of the compound HWs in Northeast China and Northwest China occurred during early summer. Most of daytime HWs concentrated in northern and western parts of China, and were inclined to appear as dry events accompanied by low relative humidity. The nighttime HWs tended to occur in South China and Southwest China. Such kind of HWs mainly covered peak-late summer, and were characterized by sharply heightened humidity level.

Compound HWs occurred more frequently with lengthening duration and greater intensity in North China, the Yangtze River Valley, South China and Southwest China. The compound HWs covered much broader regions during the past few decades, especially after 1995. The trend maps for daytime HWs showed quite contrasting patterns that less frequent, shorter-lasting and weaker events were observed in central-eastern China. The cooling of Tmax in the Huai-River Valley and increasing type transitions in other regions jointly led to decreasing spatial extents of independent daytime HWs. All the indices of nighttime HWs showed significant positive trends across China.

The above comparisons highlight that increasing HWs have taken the form of nighttime-accentuated event, typical of nighttime events and compound events respectively. Regional relative humidity changes triggered an intriguing phenomenon that dry HWs in the west became wetter and wet HWs in the east became drier, regardless of HW types. Despite consistency in trend signs among all types, the magnitude of humidity changes exhibited clear preferences, with strongest amplification detected in compound HWs.

### Discussions

Heat waves in this study were defined by the 90^th^ percentile. However, a more comprehensive study should include examination of severer HWs^33^. Given the 95^th^ percentile, the number of heat waves decreased by more than half in each type (figure omitted, Supplementary Fig. S3). With the 99^th^ percentile, most stations registered less than 10 events, and such small sample size would considerably impair the reliability of estimated trends. Linear trends of various aspects of heat waves by the 95^th^ percentile displayed highly similar patterns to those defined by the 90^th^ percentile. Particularly, the intensity showed much larger increases in severe events than that in 90^th^ percentile-based events, implying that severe events (95^th^ percentile) were getting even severer. In the operationally practice, the CMA (China Meteorological Administration) issues a heat wave warning when the Tmax above 35 °C lasts for at least 3 days. Events based on such absolute threshold preferred to occur in southern part of China and Northwest China, with an average annual occurrence of 2–4 events. Obviously, the CMA-defined heat waves are not as extreme as those defined by the 90^th^ percentile. The CMA-defined heat waves showed negative trends in central-eastern China, and positive trends along southeast coastal regions, similar to the pattern shown in daytime events.

Regarding mechanisms for HWs, long-lived anticyclones are deemed most responsible^34^. More specifically, the configuration between the East Asia jet stream and the western Pacifica subtropical high determine the location and intensity of heat waves in southern parts of China^35^. In northern parts of China, positive trends of compound and daytime events are more ascribed to increasing occurrence of the anticyclone residing over the Lake Baikal^13^. No matter within the anticyclone or in the secondary circulation cells around jets, resulting descending motion is critical in triggering HWs. It, on one hand, induces strong adiabatic heating near the surface; on the hand, it disperses widespread clouds, so more solar radiation can reach the surface. The above processes contribute to extreme Tmax and daytime events^13^. For nighttime HWs, moisture transport by favorable synoptic patterns is also imperative (Fig. 2)^16^. Nevertheless, the moisture source during nighttime and compound events in China remains unknown. The question of what differences in circulation patterns distinguish the formation of compound events and individual daytime/nighttime events needs to be addressed. Why strongest changes in relative humidity prefer to accompany with compound events is worth further exploring.

In addition to observed trends, projected changes in different HW types are also important. Model projections could answer whether the observed trends will continue or even accelerate. Projected results may also confirm the dominant heat wave type in the future. Further attribution of these observed and projected trends would help assess relative contributions from anthropogenic effects (e.g. urbanization, greenhouse gas, and aerosols) and natural variabilities to HW changes^36^, both of which may also vary among different types of heat waves.

Our follow-up studies would concern on dynamic mechanisms, projections and attributions of different HW types in China. Relevant conclusions would yield a full understanding of heat wave changes in China.

## Data and Methods

### Data

To define and measure different types of HWs, we use daily maximum temperature (Tmax), daily minimum temperature (Tmin), daily mean temperature (Tmean) and daily mean relative humidity observed at 756 stations in China during 1961–2010. This dataset is gained from the National Meteorological Information Center (NMIC). Before releasing the dataset, the NMIC performed rigorous quality control procedures, including correcting suspect/wrong observations and rectifying inhomogeneities possibly caused by new instrumentations and site relocations (documentations available online http://cdc.cma.gov.cn/home.do). Having been utilized in many studies, it is deemed one of the best daily datasets in China currently available^37^,^38^. This study focuses on summer period spanning from June-August, since HWs during this season are of the highest impacts. Apart from the quality control procedures by the NMIC, we also require that:

(1) missing values in each variable account for less than 5% of total records in each summer;

(2) the horizontal relocation is less than 20 km and the elevation change is less than 50 m during 1961–2010.

Based on the density of observation network, each station is representative of the domain within 25–30 km-radius around it. The threshold of 20 km is therefore routinely required for this dataset to eliminate inhomogeneous stations experiencing significant displacements^39^.

Through above procedures, 376 stations are retained. Basically, these stations are well-distributed in eastern China (Fig. 1). In western parts, however, there are far fewer stations dotted.

### Definition

A heat wave is defined as a period of at least three consecutive hot days or/and hot nights. A hot day/night refers to the one with its Tmax/Tmin exceeding long-term (1961–2010) daily 90^th^ percentiles. As recommended by Della-Marta *et al*.^40^, for each calendar day, its daily 90^th^ percentile of Tmax/Tmin is calculated based on 15-day samples centered on this day (i.e. total samples 15 × 50 = 750 days during 1961–2010). The 90^th^ percentile is a proper compromise between enough HW occurrences and event extremity^3^,^41^.

Specifically, daytime HW, nighttime HW and compound HW are defined as follows:

(1) daytime HW- at least three consecutive hot days without any accompanying hot nights (i.e. Tmax ≥ 90^th^ percentile, and Tmin < 90^th^ percentile);

(2) nighttime HW- at least three consecutive hot nights without any accompanying hot days (i.e. Tmin ≥ 90^th^ percentile, and Tmax < 90^th^ percentile);

(3) compound HW- at least three consecutive days with simultaneous hot days and hot nights (i.e. Tmax ≥ 90^th^ percentile, and Tmin ≥ 90^th^ percentile).

### Indices

In the context of above definitions, several indices are constructed to measure diverse aspects of heat waves, including:

Heat wave number (HWN) – the number of HW occurrences;

Heat wave day frequency (HWF) – the number of participating days contained in all HWs

Heat wave duration (HWD) – the mean length of HWs;

Heat wave intensity (HWI) – the amplitude of HW, calculated as accumulated exceedances above the threshold during each HW.

The HWN considers the heat wave as an integral process, while the HWF counts the total number of participating days/nights. For example, assuming 2 heat waves in a year with one lasting for 4 days and the other lasting for 6 days, corresponding HWN is 2 and the HWF is 10 days. Derived annual mean HWD is 5 days. HWI reflects accumulated heat stresses during HWs. Longer duration and larger exceedance contribute to greater HWI. The HWI is expressed in detail as follows:


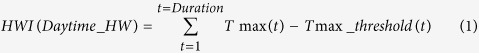



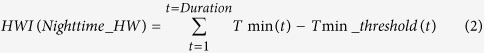



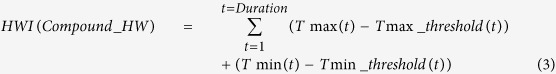


The spatial extent is another important indicator depicting the impact of HWs, especially for energy sectors. A “frozen grid” scheme, which takes full advantages of real observations, is adopted to construct scale index^42^. Specifically, the mainland of China is divided into several 2.5° longitude ×2.5° latitude boxes. It is assumed that totally ns(*i*) stations are located within box-*i*; among these ns(*i*) stations, nh(*i,t*) stations observe at least one heat wave during summer in year-*t*. Then the spatial coverage of HWs in year-*t* is computed as:


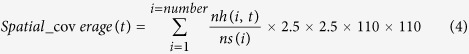


in which “110” denotes the approximate distance per unit longitude/latitude, and “number” denotes the total number of boxes covering China. This scheme impartially integrates contributions to total spatial coverage from different sub-regions, regardless of their uneven station distributions. Sensitive tests indicate that the selection of 2°, 2.5°and 3° for the box scale yields highly similar results, and doesn’t influence the following trend analyses significantly.

### Slope estimator

Linear trends of various indices are evaluated via the Kendall’s tau slope estimator. This nonparametric method does not pre-assume any distributional forms of raw data and is robust against potential outliers^27^. As suggested by Perkins and Alexander^3^, trends are computed at a station only when at least 20 years of events exist, and statistical significance is set at the 0.05 level.

## Additional Information

**How to cite this article:** Chen, Y. and Li, Y. An Inter-comparison of Three Heat Wave Types in China during 1961–2010 Observed Basic Features and Linear Trends. *Sci. Rep.* **7**, 45619; doi: 10.1038/srep45619 (2017).

**Publisher's note:** Springer Nature remains neutral with regard to jurisdictional claims in published maps and institutional affiliations.

## Supplementary Material

Supplementary Figures
