# Supplementary material for: An Inter-comparison of Three Heat Wave Types in China during 1961–2010: Observed Basic Features and Linear Trends
Source: Sci Rep. 2017 Mar 31;7:45619. doi: 10.1038/srep45619 (PMC5374538; doi:10.1038/srep45619)
Supplement: Supplementary Figures [file srep45619-s1.pdf]

An Inter-comparison of Three Heat Wave Types in China during 1961-2010: Observed Basic Features and Linear Trends

Yang Chen[1,*], and Yi Li[1,2]

[1] Chinese Academy of Meteorological Sciences, State Key Laboratory of Severe Weather, Beijing, 100081, China

[2] National Climate Center of China, Laboratory of Climate Studies, Beijing, 100081, China

*Corresponding to wawywawl@163.com

Supplementary Figures Submitted to Scientific Reports

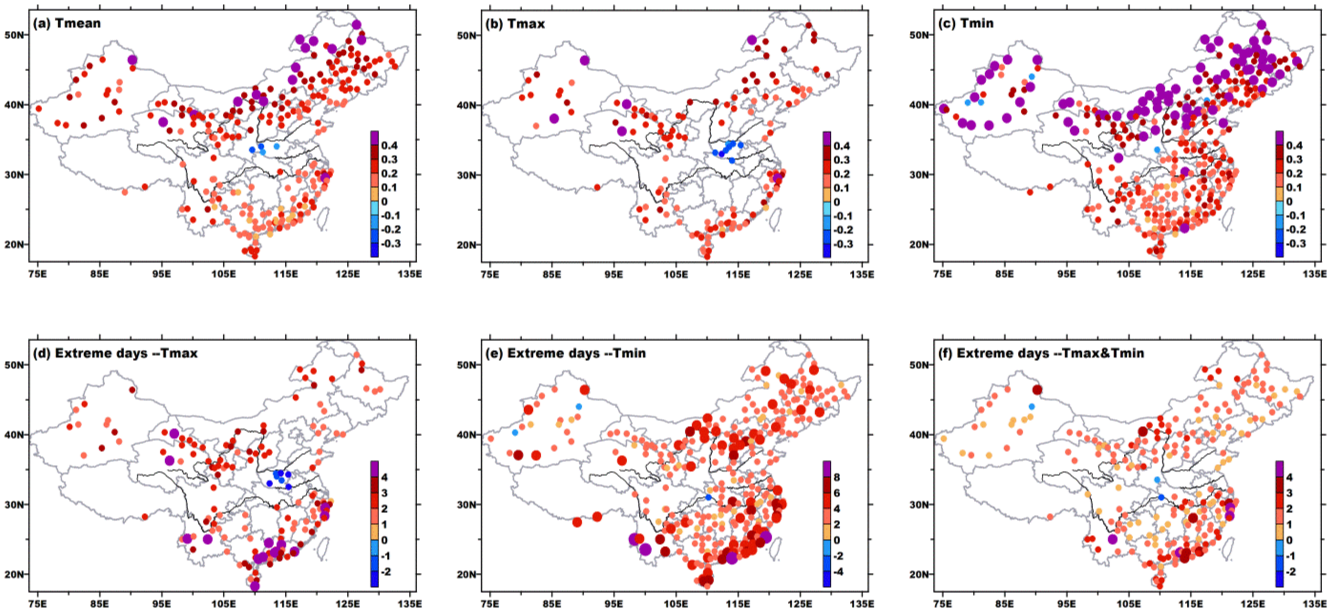

Figure S1. Linear trends (℃ decade^{-1} for a-c, days decade^{-1} for d-e) of daily mean temperature (a), maximum temperature (b), minimum temperature (c), extreme warm days (d, only Tmax>=90th percentile), extreme warm nights (e, only Tmin>=90th percentile), and extreme compound events (f, Tmax>=90th percentile and Tmin>=90th percentile). Significant and insignificant trends at the 0.05 level are denoted by shaded and blank dots, respectively. This figure was created by Surfer 7.0 (http://www.goldensoftware.com/products/surfer).

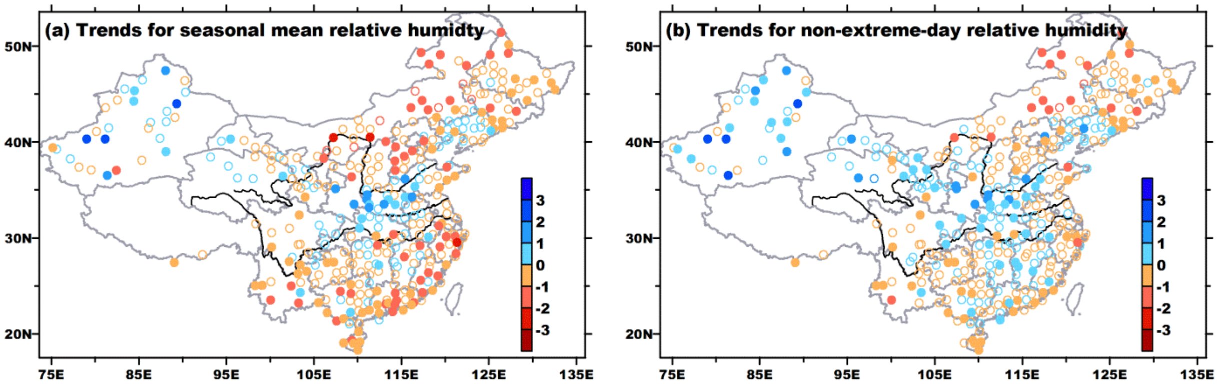

Figure S2. Linear trends of relative humidity (% decade^{-1}) for (a) summer-mean values and (b) non-extreme-day mean values. Non-extreme-day refers to the day with both Tmax and Tmin below their respective 90th percentiles. Significant and insignificant trends are denoted by shaded and blank dots, respectively. This figure was created by Surfer 7.0 (http://www.goldensoftware.com/products/surfer).

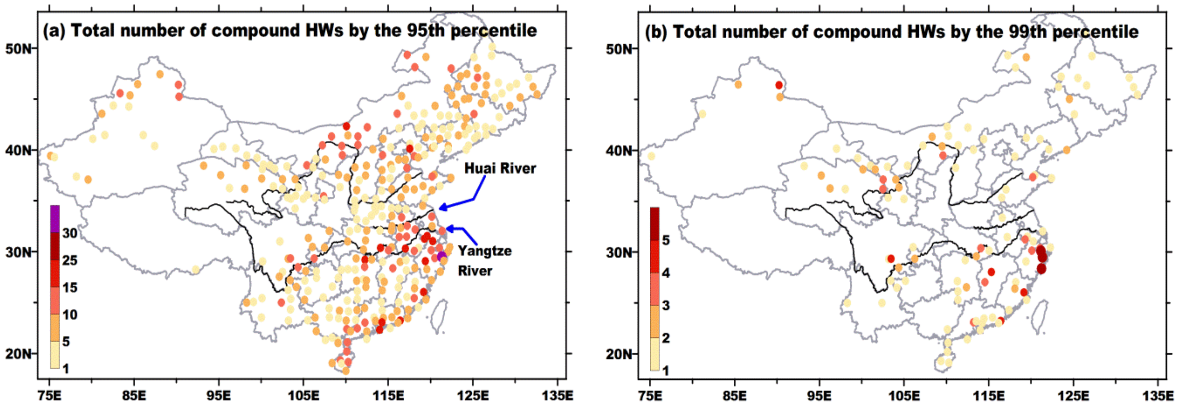

Figure S3 Accumulated frequency of heat waves by the 95th (a) and 99th (b) percentiles during 1961-2010. This figure was created by Surfer 7.0 (http://www.goldensoftware.com/products/surfer).
